# The genome sequence of the Taiga Bean Goose, *Anser fabalis* (Latham, 1787) (Anseriformes: Anatidae)

**DOI:** 10.12688/wellcomeopenres.26717.1

**Published:** 2026-06-02

**Authors:** Hannah C. Staniforth, Michelle F. O’Brien, Rosa Lopez Colom, Natural History Museum Genome Acquisition Lab, Wellcome Sanger Institute Tree of Life Management, Samples and Laboratory team, Wellcome Sanger Institute Scientific Operations: Sequencing Operations, Wellcome Sanger Institute Tree of Life Core Informatics team, Tree of Life Core Informatics collective, Darwin Tree of Life Consortium

**Affiliations:** 1 Wildfowl and Wetlands Trust, Slimbridge, England, UK

**Keywords:** Anser fabalis, Taiga Bean Goose, genome sequence, chromosomal, Anseriformes

## Abstract

We present a genome assembly from an individual male *Anser fabalis* (Taiga Bean-Goose; Chordata; Aves; Anseriformes; Anatidae). The genome sequence has a total length of 1 316.27 megabases. Most of the assembly (87.89%) is scaffolded into 38 chromosomal pseudomolecules, including the Z sex chromosome. The mitochondrial genome has also been assembled, with a length of 16.74 kilobases. This assembly was generated as part of the Darwin Tree of Life project, which produces genomes for eukaryotic species found in Britain and Ireland.

## Species taxonomy

Eukaryota; Metazoa; Chordata; Aves; Anseriformes; Anatidae; *Anser*; *Anser fabalis* (Latham, 1787) (NCBI:txid132587).

## Background

The Taiga Bean Goose ( *Anser fabalis fabalis*) is a migratory species of grey goose with a large geographical range across northern Eurasia. One of the larger species in the grey goose genus, it has a wingspan of 147–157 cm and weight range of 2.2–4.0 kg ( RSPB, 2025). Adult plumage is brown-grey over the body and wings. The head is dark brown and relatively small with a slender, black and orange bill. A large portion of the bill is orange and this distinguishes it from the Tundra Bean Goose ( *Anser fabalis rossicus/Anser serrirostris*), which has a single band of orange on the bill ( AEWA, 2025a). Their legs and feet are uniformly orange. *Anser fabalis* has been the subject of taxonomic debate, and no agreement has been reached about the number of subspecies ( Ottenburghs *et al.*, 2020).

The species has a broad range over much of northern Eurasia. Its name derives from the boreal taiga forests which make up its breeding grounds, stretching through Northern Europe, Siberia and Mongolia to the Sea of Okhotsk ( Kirwan *et al.*, 2024). The name “Bean Goose” was coined to reflect its preference for grazing Field Bean ( *Vicia faba*), which used to be more widely farmed ( Owen & Burn, 1977). Their diet consists primarily of grasses, sedges and moss, supplemented with berries during the breeding season and agricultural grain and sprouting cereal crops over winter ( BirdLife International, 2018a).

Populations breed in early May before moving north to congregate in large moulting flocks, where they remain flightless from July to August ( BirdLife International, 2018b). Post moult in early September, groups will migrate south and west or eastwards, to non-breeding grounds in western and Central Europe and east Asia (China, Korea, Japan) for the winter, arriving through late September ( Kirwan *et al.*, 2024).

During the breeding season, the taiga forest zone provides dense spruce with bogs and mires, the species has shown preference for scrubby birch *Betula* spp. habitat ( BirdLife International, 2018a). Nests are found within 400 m of open mire and 2 km of open water, typically in bog land on raised hummocks. For wintering grounds and migratory passage they seek arable and pastureland or marshes, including rice paddies, damp steppe grassland, open floodlands and coastal shallows ( BirdLife International, 2018a).

Age of breeding is observed to be 3 to 4 years old, and for those that reach maturity, the typical life expectancy is 7 years ( Owen & Burn, 1977). A typical clutch size is 4 to 6 eggs with the female bird incubating the nest for 27 to 29 days ( British Trust for Ornithology, 2025).

The IUCN categorises the Taiga Bean Goose as a species of least concern. Threats to population decline centre mostly on habitat degradation and human persecution ( BirdLife International, 2018a). This categorisation is based on the populations of both Taiga Bean and Tundra Bean goose, the latter having a stable population ( AEWA, 2025b). The Taiga Bean Goose is known to have a declining population with the winter population declining from an estimated 100 000 in the mid-1990s to 63 000 in 2009 ( AEWA, 2025b). The African-Eurasian Migratory Waterbird Agreement (AEWA) has formed an ‘International Single Species Action Plan’ as part of its European Goose Management Platform for the Taiga Bean Goose. This action plan covers four major subpopulations of Taiga Bean Goose in geographic regions known for wintering and breeding.

We present a chromosome-level genome sequence for *Anser fabalis.* Another scaffold-level assembly for this species is also available (GCA_056577465.1), submitted by Bird 10,000 Genomes (B10K) Consortium (data obtained via NCBI datasets, O’Leary *et al.*, 2024).

## Methods

### Sample acquisition

**Figure 1.**
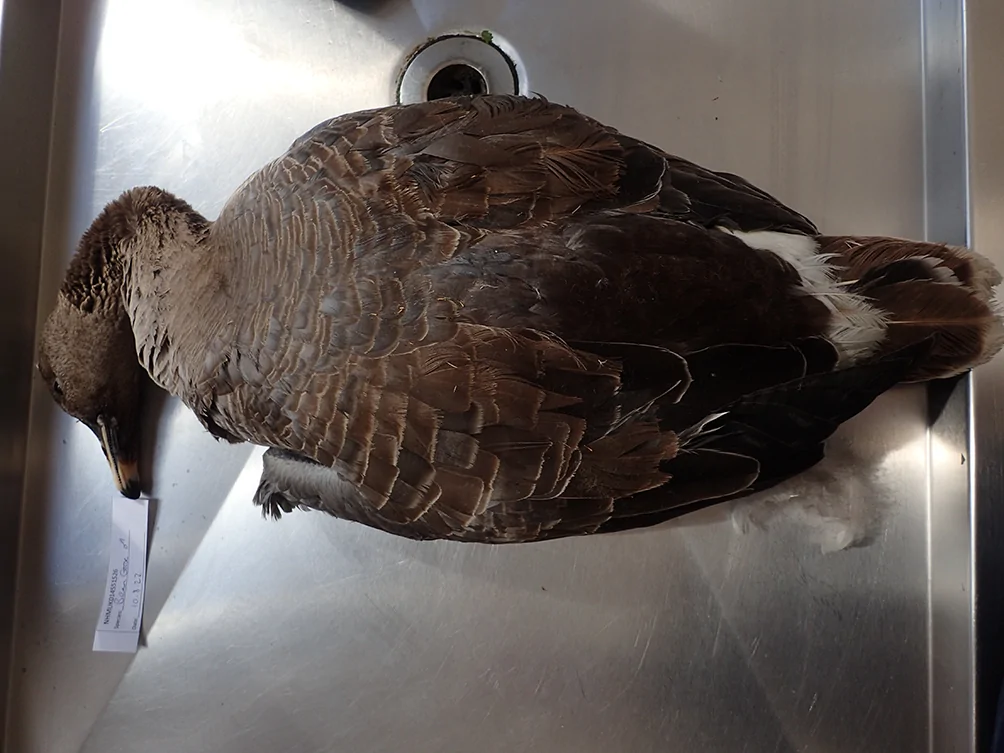
Photograph of the *Anser fabalis* (bAnsFab1) specimen used for genome sequencing.

The specimen used for genome sequencing was an adult male captive deceased specimen of *Anser fabalis* (specimen ID NHMUK014551526, ToLID bAnsFab1; Figure 1), collected from Newgounds, Gloucester, England, UK (latitude 51.73, longitude −2.40) on 2022-08-10. The specimen was collected and identified by Michelle O’Brien (Wildfowl & Wetlands Trust). Several small samples of pectoral muscle were taken and stored at −20 °C. The same specimen was used for RNA sequencing.

### Nucleic acid extraction

Detailed protocols for nucleic acid extraction developed at the Wellcome Sanger Institute (WSI) Tree of Life Core Laboratory are available on protocols.io ( Howard *et al.*, 2025). The bAnsFab1 sample was weighed and triaged to determine the appropriate extraction protocol. For HMW DNA extraction, 58.0 mg of muscle tissue was used. The tissue was homogenised by cryogenic disruption using the Covaris cryoPREP® Automated Dry Pulverizer. High molecular weight (HMW) DNA was extracted in the WSI Scientific Operations core using the Automated MagAttract v2 protocol. DNA was sheared into an average fragment size of 12–20 kb following the Megaruptor®3 for LI PacBio protocol. Sheared DNA was purified by manual SPRI (solid-phase reversible immobilisation). The concentration of the sheared and purified DNA was assessed using a Nanodrop spectrophotometer and Qubit Fluorometer using the Qubit dsDNA High Sensitivity Assay kit. Fragment size distribution was evaluated by running the sample on the FemtoPulse system. For this sample, the final post-shearing DNA had a Qubit concentration of 76.4 ng/μL and a yield of 671.00 ng, with a fragment size of 11.6 kb.

RNA was extracted from muscle tissue of bAnsFab1 in the Tree of Life Laboratory at the WSI using the RNA Extraction: Automated MagMax™ *mir*Vana protocol. The RNA concentration was assessed using a Nanodrop spectrophotometer and a Qubit Fluorometer using the Qubit RNA Broad-Range Assay kit. Analysis of the integrity of the RNA was done using the Agilent RNA 6000 Pico Kit and Eukaryotic Total RNA assay.

### PacBio HiFi library preparation and sequencing

Library preparation and sequencing were performed at the WSI Scientific Operations core. Libraries were prepared using the SMRTbell Prep Kit 3.0 (Pacific Biosciences, California, USA), following the manufacturer’s instructions. The kit includes reagents for end repair/A-tailing, adapter ligation, post-ligation SMRTbell bead clean-up, and nuclease treatment. Size selection and clean-up were performed using diluted AMPure PB beads (Pacific Biosciences). DNA concentration was quantified using a Qubit Fluorometer v4.0 (ThermoFisher Scientific) and the Qubit 1X dsDNA HS assay kit. Final library fragment size was assessed with the Agilent Femto Pulse Automated Pulsed Field CE Instrument (Agilent Technologies) using the gDNA 55 kb BAC analysis kit.

The sample was sequenced on a Revio instrument (Pacific Biosciences). The prepared library was normalised to 2 nM, and 15 μL was used for making complexes. Primers were annealed and polymerases bound to generate circularised complexes, following the manufacturer’s instructions. Complexes were purified using 1.2X SMRTbell beads, then diluted to the Revio loading concentration (200–300 pM) and spiked with a Revio sequencing internal control. The sample was sequenced on a Revio 25 M SMRT cell. The SMRT Link software (Pacific Biosciences), a web-based workflow manager, was used to configure and monitor the run and to carry out primary and secondary data analysis.

### Hi-C

***Sample preparation and crosslinking***

The Hi-C sample was prepared from 20–50 mg of frozen muscle tissue from the bAnsFab1 sample using the Arima-HiC v2 kit (Arima Genomics). Following the manufacturer’s instructions, tissue was fixed and DNA crosslinked using TC buffer to a final formaldehyde concentration of 2%. The tissue was homogenised using the Diagnocine Power Masher-II. Crosslinked DNA was digested with a restriction enzyme master mix, biotinylated, and ligated. Clean-up was performed with SPRISelect beads before library preparation. DNA concentration was measured with the Qubit Fluorometer (Thermo Fisher Scientific) and Qubit HS Assay Kit. The biotinylation percentage was estimated using the Arima-HiC v2 QC beads.

***Hi-C library preparation and sequencing***

Biotinylated DNA constructs were fragmented using a Covaris E220 sonicator and size selected to 400–600 bp using SPRISelect beads. DNA was enriched with Arima-HiC v2 kit Enrichment beads. End repair, A-tailing, and adapter ligation were carried out with the NEBNext Ultra II DNA Library Prep Kit (New England Biolabs), following a modified protocol where library preparation occurs while DNA remains bound to the Enrichment beads. Library amplification was performed using KAPA HiFi HotStart mix and a custom Unique Dual Index (UDI) barcode set (Integrated DNA Technologies). Depending on sample concentration and biotinylation percentage determined at the crosslinking stage, libraries were amplified with 10–16 PCR cycles. Post-PCR clean-up was performed with SPRISelect beads. Libraries were quantified using the AccuClear Ultra High Sensitivity dsDNA Standards Assay Kit (Biotium) and a FLUOstar Omega plate reader (BMG Labtech).

Prior to sequencing, libraries were normalised to 10 ng/μL. Normalised libraries were quantified again to create equimolar and/or weighted 2.8 nM pools. Pool concentrations were checked using the Agilent 4200 TapeStation (Agilent) with High Sensitivity D500 reagents before sequencing. Sequencing was performed using paired-end 150 bp reads on the Illumina NovaSeq X.

### RNA library preparation and sequencing

Libraries were prepared using the NEBNext® Ultra™ II Directional RNA Library Prep Kit for Illumina (New England Biolabs), following the manufacturer’s instructions. Poly(A) mRNA in the total RNA solution was isolated using oligo (dT) beads, converted to cDNA, and uniquely indexed; 14 PCR cycles were performed. Libraries were size-selected to produce fragments between 100–300 bp. Libraries were quantified, normalised, pooled to a final concentration of 2.8 nM, and diluted to 150 pM for loading. Sequencing was carried out on the Illumina NovaSeq X, generating paired-end reads.

### Genome assembly

Prior to assembly of the PacBio HiFi reads, a database of *k*-mer counts ( *k* = 31) was generated from the filtered reads using FastK. GenomeScope2 ( Ranallo-Benavidez *et al.*, 2020) was used to analyse the *k*-mer frequency distributions, providing estimates of genome size, heterozygosity, and repeat content.

The HiFi reads were assembled using Hifiasm ( Cheng *et al.*, 2021) with the --primary option. Haplotypic duplications were identified and removed using purge_dups ( Guan *et al.*, 2020). The Hi-C reads ( Rao *et al.*, 2014) were mapped to the primary contigs using bwa-mem2 ( Vasimuddin *et al.*, 2019), and the contigs were scaffolded in YaHS ( Zhou *et al.*, 2023) with the --break option for handling potential misassemblies. The scaffolded assemblies were evaluated using Gfastats ( Formenti *et al.*, 2022), BUSCO ( Manni *et al.*, 2021) and MerquryFK ( Rhie *et al.*, 2020).

The mitochondrial genome was assembled using OATK ( Zhou *et al.*, 2025).

### Assembly curation

The assembly was decontaminated using the Assembly Screen for Cobionts and Contaminants ( ASCC) pipeline. TreeVal was used to generate the flat files and maps for use in curation. Manual curation was conducted primarily in PretextView and HiGlass ( Kerpedjiev *et al.*, 2018). Scaffolds were visually inspected and corrected as described by Howe *et al*. (2021). Manual corrections included six breaks and 23 joins. This reduced the scaffold count by 1.7% and increased the scaffold N50 by 26.4%. The curation process is described at https://gitlab.com/wtsi-grit/rapid-curation . PretextSnapshot was used to generate a Hi-C contact map of the final assembly.

### Assembly quality assessment

The MerquryFK tool ( Rhie *et al.*, 2020) was run in a Singularity container ( Kurtzer *et al.*, 2017) to evaluate *k*-mer completeness and assembly quality for the primary and alternate haplotypes using the *k*-mer database ( *k* = 31) computed prior to genome assembly. The analysis outputs included assembly QV scores and completeness statistics.

The genome was analysed using the BlobToolKit pipeline, a Nextflow implementation of the earlier Snakemake version ( Challis *et al.*, 2020). The pipeline aligns PacBio reads using minimap2 ( Li, 2018) and SAMtools ( Danecek *et al.*, 2021) to generate coverage tracks. It runs BUSCO ( Manni *et al.*, 2021) using lineages identified from the NCBI Taxonomy ( Schoch *et al.*, 2020). For the three domain-level lineages, BUSCO genes are aligned to the UniProt Reference Proteomes database ( Bateman *et al.*, 2023) using DIAMOND blastp ( Buchfink *et al.*, 2021). The genome is divided into chunks based on the density of BUSCO genes from the closest taxonomic lineage, and each chunk is aligned to the UniProt Reference Proteomes database with DIAMOND blastx. Sequences without hits are chunked using seqtk and aligned to the NT database with blastn ( Altschul *et al.*, 1990). The BlobToolKit suite consolidates all outputs into a blobdir for visualisation. The BlobToolKit pipeline was developed using nf-core tooling ( Ewels *et al.*, 2020) and MultiQC ( Ewels *et al.*, 2016), with containerisation through Docker ( Merkel, 2014) and Singularity ( Kurtzer *et al.*, 2017).

## Genome sequence report

### Sequence data

**Figure 2.**
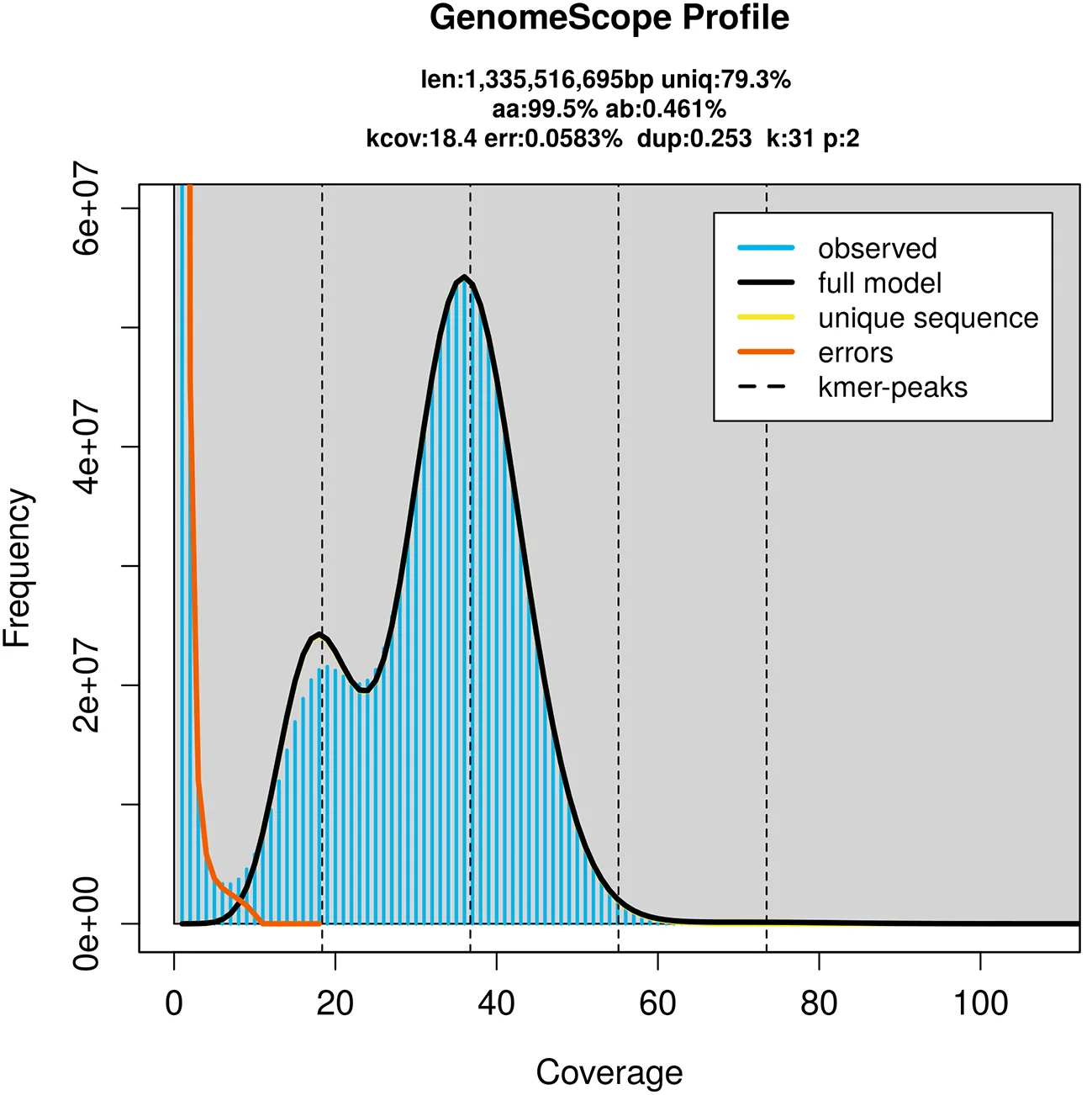
Frequency distribution of *k*-mers generated using GenomeScope2. The plot shows observed and modelled *k*-mer spectra, providing estimates of genome size, heterozygosity, and repeat content based on unassembled sequencing reads.

PacBio sequencing of the *Anser fabalis* specimen generated 50.13 Gb (gigabases) from 5.65 million reads, which were used to assemble the genome. GenomeScope2.0 analysis estimated the haploid genome size at 1 335.86 Mb, with a heterozygosity of 0.46% and repeat content of 20.72% ( Figure 2). These estimates guided expectations for the assembly. Based on the estimated genome size, the sequencing data provided approximately 37× coverage. Hi-C sequencing produced 43.54 Gb from 144.19 million reads, which were used to scaffold the assembly. RNA sequencing data were also generated and are available in public sequence repositories. Table 1 summarises the specimen and sequencing details.

**Table 1.** Specimen and sequencing data for *Anser fabalis* (BioProject PRJEB75623).

Platform	PacBio HiFi	Hi-C	RNA-seq
**ToLID**	bAnsFab1	bAnsFab1	bAnsFab1
**Specimen ID**	NHMUK014551526	NHMUK014551526	NHMUK014551526
**BioSample (source individual)**	SAMEA113398846	SAMEA113398846	SAMEA113398846
**BioSample (tissue)**	SAMEA113398919	SAMEA113398919	SAMEA113398919
**Tissue**	muscle	muscle	muscle
**Instrument**	Revio	Illumina NovaSeq X	Illumina NovaSeq X
**Run accessions**	ERR13112056	ERR13093677	ERR13493950
**Read count total**	5.65 million	144.19 million	33.18 million
**Base count total**	50.13 Gb	43.54 Gb	10.02 Gb

### Assembly statistics

The primary haplotype was assembled, and contigs corresponding to an alternate haplotype were also deposited in INSDC databases. The final assembly has a total length of 1 316.27 Mb in 878 scaffolds, with 973 gaps, and a scaffold N50 of 78.82 Mb ( Table 2).

**Table 2.** Genome assembly data for *Anser fabalis.*

Genome assembly	Primary assembly
**Assembly name**	bAnsFab1.1
**Assembly accession**	GCA_965250395.1
**Alternate haplotype accession**	GCA_965250455.1
**Alternate haplotype longest scaffold length (Mb)**	7.13
**Assembly level**	chromosome
**Span (Mb)**	1 316.27
**Number of chromosomes**	38
**Number of contigs**	1 851
**Contig N50**	2.2 Mb
**Number of scaffolds**	878
**Scaffold N50**	78.82 Mb
**Sex chromosomes**	Z
**Organelles**	Mitochondrion: 16.74 kb

**Figure 3.**
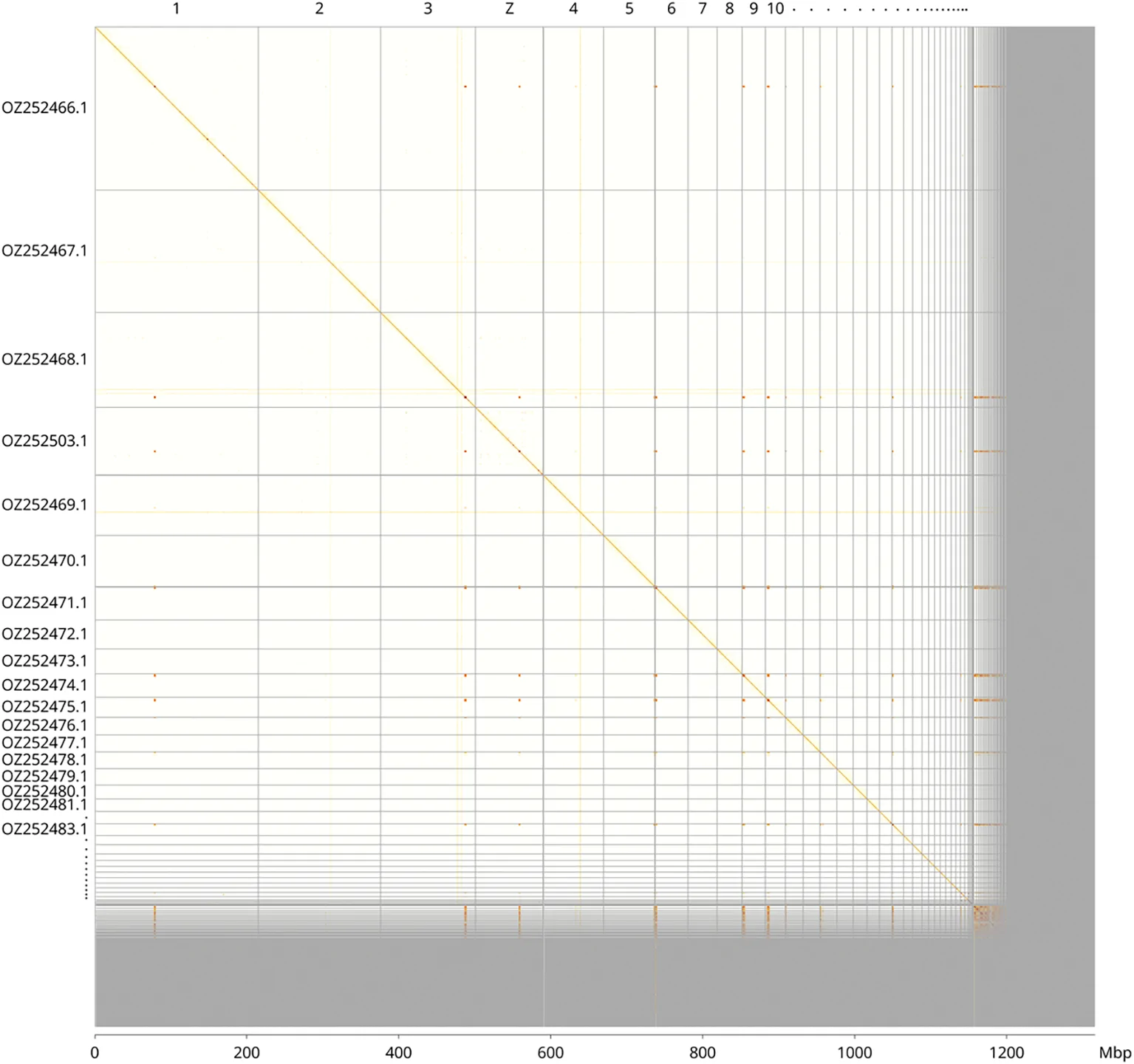
Hi-C contact map of the *Anser fabalis* genome assembly. Assembled chromosomes are shown in order of size and labelled along the axes, with a megabase scale shown below. The plot was generated using PretextSnapshot.

Most of the assembly sequence (87.89%) was assigned to 38 chromosomal-level scaffolds, representing 37 autosomes and the Z sex chromosome. These chromosome-level scaffolds, confirmed by Hi-C data, are named according to size ( Figure 3; Table 3).

**Table 3.** Chromosomal pseudomolecules in the primary genome assembly of *Anser fabalis* bAnsFab1.

INSDC accession	Molecule	Length (Mb)	GC%
OZ252466.1	1	215.30	40.50
OZ252467.1	2	161.12	40
OZ252468.1	3	124.47	41
OZ252469.1	4	78.82	40
OZ252470.1	5	68.06	42
OZ252471.1	6	42.94	42.50
OZ252472.1	7	38.49	42
OZ252473.1	8	32.88	42
OZ252474.1	9	30.57	45
OZ252475.1	10	26.34	45.50
OZ252476.1	11	23.13	43.50
OZ252477.1	12	22.53	43.50
OZ252478.1	13	21.91	46
OZ252479.1	14	21.60	43
OZ252480.1	15	18.29	45.50
OZ252481.1	16	16.44	46.50
OZ252482.1	17	16.27	46
OZ252483.1	18	15.28	49
OZ252484.1	19	12.27	49
OZ252485.1	20	12.05	47.50
OZ252486.1	21	8.52	48.50
OZ252487.1	22	7.87	50
OZ252488.1	23	7.65	51.50
OZ252489.1	24	7.19	53
OZ252490.1	25	7.03	52.50
OZ252491.1	26	6.44	53.50
OZ252492.1	27	6.09	50
OZ252493.1	28	5.50	58
OZ252494.1	29	4.42	59
OZ252495.1	30	1.80	53
OZ252496.1	31	1.63	46
OZ252497.1	32	1.45	54
OZ252498.1	33	0.66	61.50
OZ252499.1	34	0.56	55.50
OZ252500.1	35	0.49	57.50
OZ252501.1	36	0.47	61.50
OZ252502.1	37	0.41	51.50
OZ252503.1	Z	89.90	41

The mitochondrial genome was also assembled (length 16.74 kb, OZ252504.1). This sequence is included as a contig in the multifasta file of the genome submission and as a standalone record.

### Assembly quality metrics

**Figure 4.**
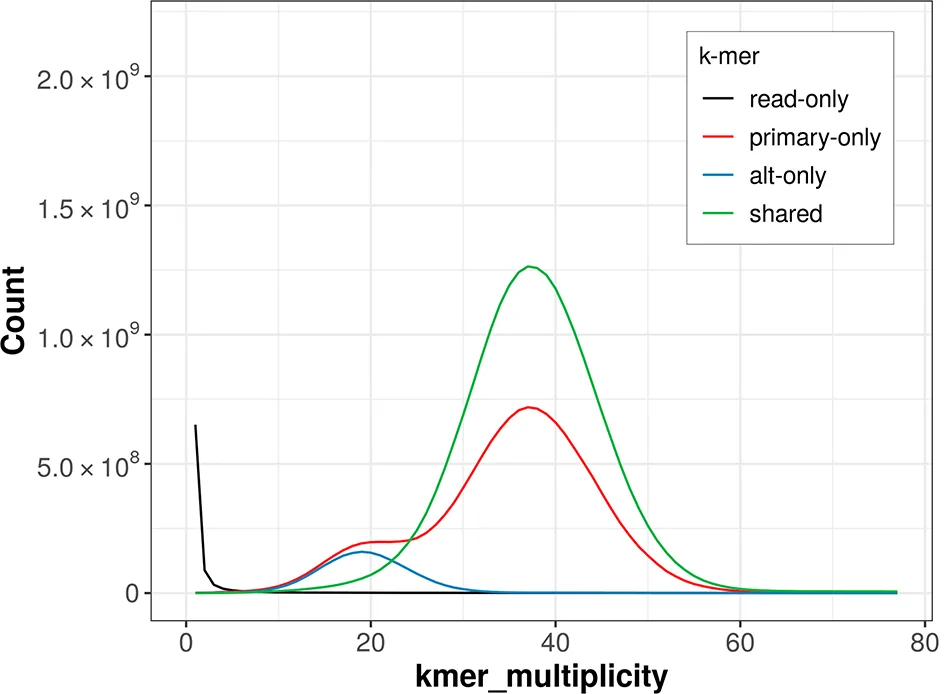
Evaluation of *k*-mer completeness using MerquryFK. This plot illustrates the recovery of *k*-mers from the original read data in the final assemblies. The horizontal axis represents *k*-mer multiplicity, and the vertical axis shows the number of *k*-mers. The black curve represents *k*-mers that appear in the reads but are not assembled. The green curve corresponds to *k*-mers shared by both haplotypes, and the red and blue curves show *k*-mers found only in one of the haplotypes.

The combined primary and alternate assemblies achieve an estimated QV of 59.5. The *k*-mer completeness is 91.06% for the primary assembly, 61.47% for the alternate haplotype, and 99.72% for the combined assemblies ( Figure 4).

**Figure 5.**
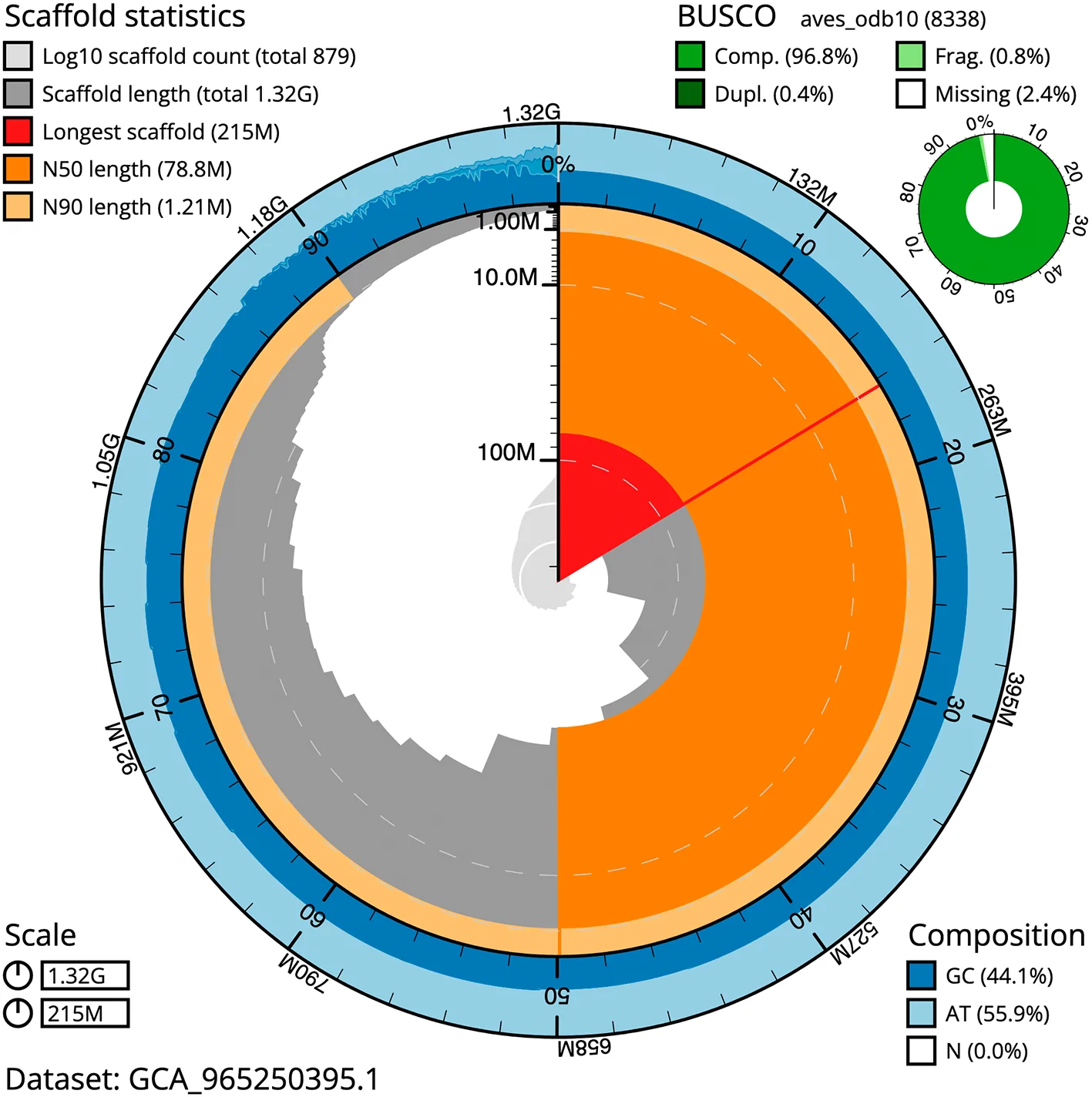
Assembly metrics for bAnsFab1.1. The BlobToolKit snail plot provides an overview of assembly metrics and BUSCO gene completeness. The circumference represents the length of the whole genome sequence, and the main plot is divided into 1 000 bins around the circumference. The outermost blue tracks display the distribution of GC, AT, and N percentages across the bins. Scaffolds are arranged clockwise from longest to shortest and are depicted in dark grey. The longest scaffold is indicated by the red arc, and the deeper orange and pale orange arcs represent the N50 and N90 lengths. A light grey spiral at the centre shows the cumulative scaffold count on a logarithmic scale. A summary of complete, fragmented, duplicated, and missing BUSCO genes in the aves_odb10 set is presented at the top right. An interactive version of this figure can be accessed on the BlobToolKit viewer.

**Figure 6.**
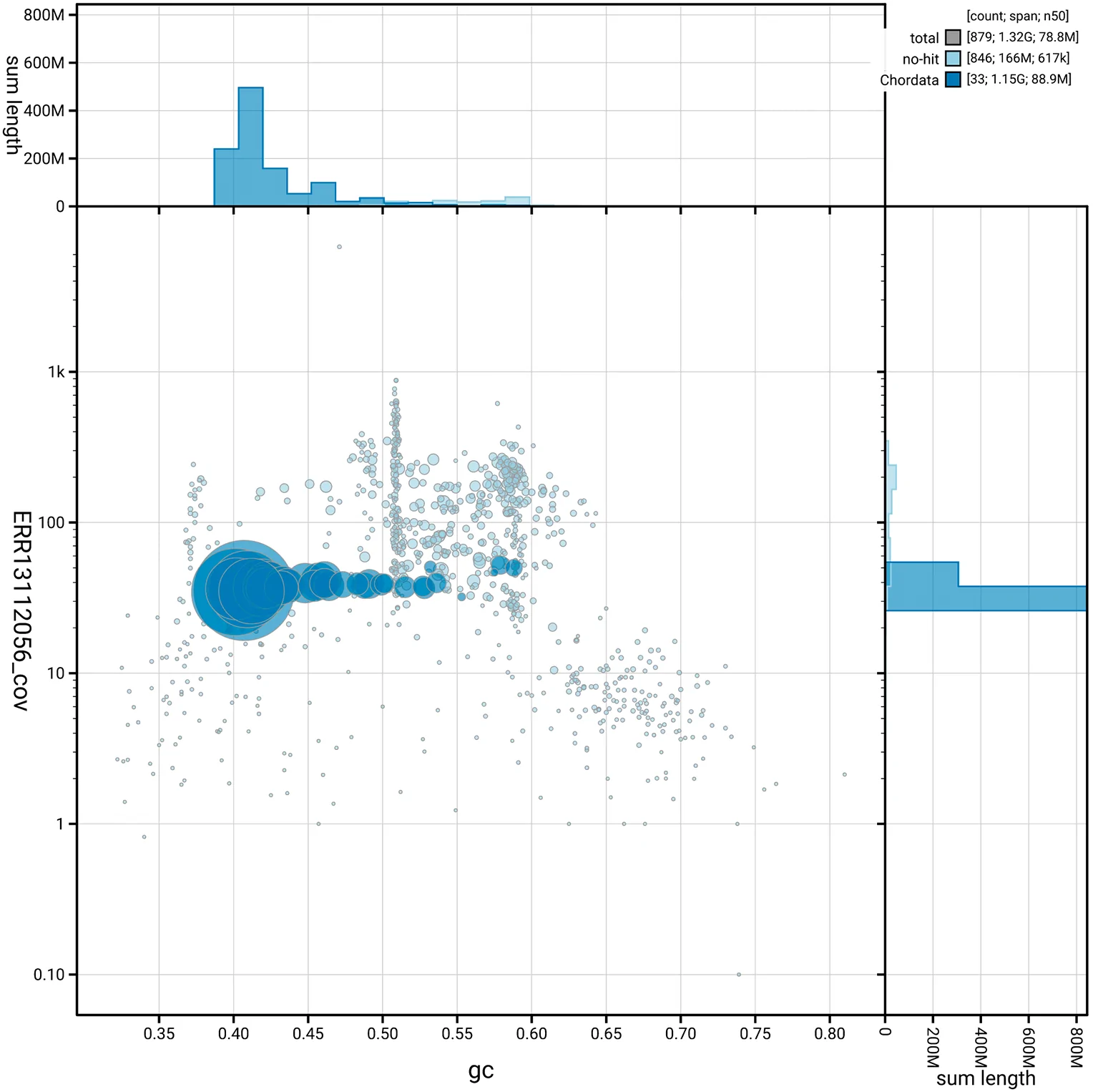
BlobToolKit blob plot for bAnsFab1.1. The plot shows base coverage (vertical axis) and GC content (horizontal axis). The circles represent scaffolds, with the size proportional to scaffold length and the colour representing phylum membership. The histograms along the axes display the total length of sequences distributed across different levels of coverage and GC content. An interactive version of this figure is available on the BlobToolKit viewer.

BUSCO v.5.7.1 analysis using the aves_odb10 reference set ( *n* = 8 338) identified 96.8% of the expected gene set (single = 96.4%, duplicated = 0.4%). The snail plot in Figure 5 summarises the scaffold length distribution and other assembly statistics for the primary assembly. The blob plot in Figure 6 shows the distribution of scaffolds by GC proportion and coverage.

Table 4 lists the assembly metric benchmarks adapted from Rhie *et al.* (2021) and the Earth BioGenome Project Report on Assembly Standards January 2026. The EBP metric, calculated for the primary assembly, is **6.7.Q59**.

**Table 4.** Earth Biogenome Project summary metrics for the *Anser fabalis* assembly.

Measure	Value	Benchmark
EBP summary (primary)	6.7.Q59	6.C.Q40
Contig N50 length	2.20 Mb	≥ 1 Mb
Scaffold N50 length	78.82 Mb	= chromosome N50
Consensus quality (QV)	Primary: 59.5; alternate: 59.5; combined: 59.5	≥ 40
*k*-mer completeness	Primary: 91.06%; alternate: 61.47%; combined: 99.72%	≥ 95%
BUSCO	C:96.8% [S:96.4%, D:0.4%], F:0.8%, M:2.4%, n:8 338	S > 90%; D < 5%
Percentage of assembly assigned to chromosomes	87.89%	≥ 90%

**Notes:** The EBP summary uses log10(Contig N50); chromosome-level (C) or log10(Scaffold N50); Q (Merqury QV). BUSCO: C = complete; S = single-copy; D = duplicated; F = fragmented; M = missing; n = orthologues.

**Table 5.** Software versions and sources used for *Anser fabalis.*

Software	Version	Source
BLAST	2.14.0	ftp://ftp.ncbi.nlm.nih.gov/blast/executables/blast+/
BlobToolKit	4.4.5	https://github.com/blobtoolkit/blobtoolkit
BUSCO	5.7.1	https://gitlab.com/ezlab/busco
bwa-mem2	2.2.1	https://github.com/bwa-mem2/bwa-mem2
DIAMOND	2.1.8	https://github.com/bbuchfink/diamond
fasta_windows	0.2.4	https://github.com/tolkit/fasta_windows
FastK	1.1	https://github.com/thegenemyers/FASTK
GenomeScope2.0	2.0.1	https://github.com/tbenavi1/genomescope2.0
Gfastats	1.3.6	https://github.com/vgl-hub/gfastats
Hifiasm	0.19.8-r603	https://github.com/chhylp123/hifiasm
HiGlass	1.13.4	https://github.com/higlass/higlass
MerquryFK	1.1.2	https://github.com/thegenemyers/MERQURY.FK
Minimap2	2.28-r1209	https://github.com/lh3/minimap2
Oatk	1.0	https://github.com/c-zhou/oatk
MultiQC	1.14; 1.17 and 1.18	https://github.com/MultiQC/MultiQC
Nextflow	24.10.4	https://github.com/nextflow-io/nextflow
PretextSnapshot	0.0.5	https://github.com/sanger-tol/PretextSnapshot
PretextView	1.0.3	https://github.com/sanger-tol/PretextView
purge_dups	1.2.3	https://github.com/dfguan/purge_dups
samtools	1.21	https://github.com/samtools/samtools
sanger-tol/ascc	0.1.0	https://github.com/sanger-tol/ascc
sanger-tol/blobtoolkit	v0.7.1	https://github.com/sanger-tol/blobtoolkit
sanger-tol/curationpretext	1.4.2	https://github.com/sanger-tol/curationpretext
Seqtk	1.3	https://github.com/lh3/seqtk
Singularity	3.9.0	https://github.com/sylabs/singularity
TreeVal	1.4.0	https://github.com/sanger-tol/treeval
YaHS	1.1a.2	https://github.com/c-zhou/yahs

## Author information

Contributors are listed at the following links:

• Members of the Natural History Museum Genome Acquisition Lab
• Members of the Wellcome Sanger Institute Tree of Life Management, Samples and Laboratory team
• Members of Wellcome Sanger Institute Scientific Operations – Sequencing Operations
• Members of the Wellcome Sanger Institute Tree of Life Core Informatics team
• Members of the Tree of Life Core Informatics collective
• Members of the Darwin Tree of Life Consortium

## Wellcome sanger institute – legal and governance

The materials that have contributed to this genome note have been supplied by a Darwin Tree of Life Partner. The submission of materials by a Darwin Tree of Life Partner is subject to the **‘Darwin Tree of Life Project Sampling Code of Practice’**, which can be found in full on the Darwin Tree of Life website. By agreeing with and signing up to the Sampling Code of Practice, the Darwin Tree of Life Partner agrees they will meet the legal and ethical requirements and standards set out within this document in respect of all samples acquired for, and supplied to, the Darwin Tree of Life Project. Further, the Wellcome Sanger Institute employs a process whereby due diligence is carried out proportionate to the nature of the materials themselves, and the circumstances under which they have been/are to be collected and provided for use. The purpose of this is to address and mitigate any potential legal and/or ethical implications of receipt and use of the materials as part of the research project, and to ensure that in doing so we align with best practice wherever possible. The overarching areas of consideration are:

• Ethical review of provenance and sourcing of the material
• Legality of collection, transfer and use (national and international)

Each transfer of samples is further undertaken according to a Research Collaboration Agreement or Material Transfer Agreement entered into by the Darwin Tree of Life Partner, Genome Research Limited (operating as the Wellcome Sanger Institute), and in some circumstances, other Darwin Tree of Life collaborators.

## Data Availability

European Nucleotide Archive: Anser fabalis (taiga bean goose). Accession number PRJEB75623. The genome sequence is released openly for reuse. The *Anser fabalis* genome sequencing initiative is part of the Darwin Tree of Life Project (PRJEB40665), the Sanger Institute Tree of Life Programme (PRJEB43745) and the Vertebrate Genomes Project (PRJNA489243). All raw sequence data and the assembly have been deposited in INSDC databases. The genome will be annotated using available RNA-Seq data and presented through the Ensembl pipeline at the European Bioinformatics Institute. Raw data and assembly accession identifiers are reported in Tables 1 and 2. Production code used in genome assembly at the WSI Tree of Life is available at https://github.com/sanger-tol . Table 5 lists software versions used in this study.
