# Supplementary material for: The genome sequence of the Taiga Bean Goose, Anser fabalis (Latham, 1787) (Anseriformes: Anatidae)
Source: Wellcome Open Res. 2026 Jun 2;11:331. [Version 1] doi: 10.12688/wellcomeopenres.26717.1 (PMC13550897; doi:10.12688/wellcomeopenres.26717.1)
Supplement: Peer review history

# Reviewer report: Reviewer response for version 1

**DOI:** 10.21956/wellcomeopenres.29419.r158277

**Published:** 2026-07-06

**Authors:** Ruy D Chacón

**Affiliations:** 1 University of São Paulo (USP), São Paulo, Brazil

The data note “ *The genome sequence of the Taiga Bean Goose, Anser fabalis (Latham, 1787) (Anseriformes: Anatidae)*,” presented by Staniforth, O’Brien, and Lopez, contributes with relevant information of this species with a declining population. The authors used a hybrid approach to map the genome as fully as possible at the chromosomal scaffold level. However, I have a few suggestions that I believe could clarify certain points in the paper.

 •    Abstract: It is currently concise and to the point. However, I suggest adding one or two sentences describing the approach and the platforms used before presenting the results.

 •    Background: This section provides a comprehensive overview of the species’ ecology and natural history. However, the transition to the goal of genome sequencing is somewhat abrupt. It would be helpful for the introduction to include a brief paragraph highlighting the importance of chromosome-level reference genomes for comparative genomics, conservation, and evolutionary studies, as well as clarifying the added value of this assembly compared to previously available genomic resources.

 •    Sample Collection: Additional information regarding sample traceability and preanalytical handling would be helpful. If this data is available, please consider including the cause of death (or whether the subject died of natural causes), the time interval between death and tissue collection, storage conditions, storage temperature, and the time elapsed before long-term storage.

 •    Genome Assembly Report: The assembly metrics are presented clearly. However, I suggest adding one or two brief sentences that briefly interpret the “ *Percentage of assembly assigned to chromosomes,*” which is slightly below the EBP benchmark.

Are sufficient details of methods and materials provided to allow replication by others?

Yes

Is the rationale for creating the dataset(s) clearly described?

Yes

Are the datasets clearly presented in a useable and accessible format?

Yes

Are the protocols appropriate and is the work technically sound?

Yes

Reviewer Expertise:

genetics; genomics; veterinary research

I confirm that I have read this submission and believe that I have an appropriate level of expertise to confirm that it is of an acceptable scientific standard.

# Reviewer report: Reviewer response for version 1

**DOI:** 10.21956/wellcomeopenres.29419.r158285

**Published:** 2026-06-25

**Authors:** Aja Noersgaard Buur Tengstedt

**Affiliations:** 1 Aarhus University, Aarhus, Denmark

The data note presents a chromosome-level genome assembly for the Taiga Bean Goose, *Anser fabalis.*

 Overall, this is a well-prepared genome resource paper. The manuscript is clearly written, the assembly was generated using current best-practice approaches, methods are described in sufficient detail, and the underlying data appear to be publicly available.

 The resulting assembly metrics are clearly presented and indicate a high-quality genome assembly.

 Minor comments:

 1. The manuscript notes the existence of a previous scaffold-level assembly. It may be useful to include a brief comparison of assembly metrics between the previous assembly and the assembly presented here.

 2. Table 4 reports that 87.89% of the assembly sequence is assigned to chromosomes, slightly below the >90% benchmark listed in the same table. Although the difference is small, a brief discussion of the nature of the unplaced scaffolds would be appropriate.

Are sufficient details of methods and materials provided to allow replication by others?

Yes

Is the rationale for creating the dataset(s) clearly described?

Yes

Are the datasets clearly presented in a useable and accessible format?

Yes

Are the protocols appropriate and is the work technically sound?

Yes

Reviewer Expertise:

My expertise is in genomics and genome-scale data analysis. I evaluated the manuscript primarily with respect to assembly quality, methodology, and data accessibility.

I confirm that I have read this submission and believe that I have an appropriate level of expertise to confirm that it is of an acceptable scientific standard.
